# Supplementary material for: Sex and regional effects of Bacteroides in the gut
Source: mBio. 2026 May 6;17(6):e00469-26. doi: 10.1128/mbio.00469-26 (PMC13251366; doi:10.1128/mbio.00469-26)
Supplement: Supplementary file 2 [file mbio.00469-26-s0003.pdf]

Table S2: Number of genes differentially expressed by *Bacteroides* across the gut[a].

	Small Intestine	Cecum	Proximal Colon	Distal Colon
Treatment	60	55	23	26
Sex	N/A	122	23	13
Combined Effect	N/A	31	0	10
Top Pathways Regulated	Chemokine Signaling, TCR Signaling, NK-kappaB Signaling	Myeloid Activation, NLR Signaling, Coagulation	NK Activity, Oxidative Stress Response, RNA Sensing	Myeloid Activation, Chemokine Signaling, BCR Signaling

[a]For each region, genes significantly differentially expressed in each of the treatment groups of mice gavaged with PBS or Bacteroides were identified. Significantly differentially regulated genes were determined by performing a mixed-effects model on the log_2 transformed counts of each gene within each region, accounting for treatment, sex, and the combined effect of sex and treatment. Total number of genes found to be significantly affected by treatment, sex, and the combined effect of treatment and sex are listed in this table. Additionally, pathways that describe the function of each region's top 3 differentially expressed genes are highlighted in the last row.
